# GYY4137-Derived Hydrogen Sulfide Donates Electrons to the Mitochondrial Electron Transport Chain via Sulfide: Quinone Oxidoreductase in Endothelial Cells

**DOI:** 10.3390/antiox12030587

**Published:** 2023-02-27

**Authors:** Bastiaan S. Star, Elisabeth C. van der Slikke, Céline Ransy, Alain Schmitt, Robert H. Henning, Frédéric Bouillaud, Hjalmar R. Bouma

**Affiliations:** 1 Department of Clinical Pharmacy and Pharmacology, University Medical Center Groningen, University of Groningen, 9700 RB Groningen, The Netherlands; 2 The National Center for Scientific Research (CNRS), The National Institute of Health and Medical Research (Inserm), Université de Paris, F-75014 Paris, France; 3 Department of Internal Medicine, University Medical Center Groningen, University of Groningen, 9700 RB Groningen, The Netherlands

**Keywords:** mitochondria, hydrogen sulfide, GYY4137, endothelial cells, sulfide:quinone oxidoreductase

## Abstract

The protective effects of hydrogen sulphide (H_2_S) to limit oxidative injury and preserve mitochondrial function during sepsis, ischemia/reperfusion, and neurodegenerative diseases have prompted the development of soluble H_2_S-releasing compounds such as GYY4137. Yet, the effects of GYY4137 on the mitochondrial function of endothelial cells remain unclear, while this cell type comprises the first target cell after parenteral administration. Here, we specifically assessed whether human endothelial cells possess a functional sulfide:quinone oxidoreductase (SQOR), to oxidise GYY4137-released H_2_S within the mitochondria for electron donation to the electron transport chain. We demonstrate that H_2_S administration increases oxygen consumption by human umbilical vein endothelial cells (HUVECs), which does not occur in the SQOR-deficient cell line SH-SY5Y. GYY4137 releases H_2_S in HUVECs in a dose- and time-dependent fashion as quantified by oxygen consumption and confirmed by lead acetate assay, as well as AzMC fluorescence. Scavenging of intracellular H_2_S using zinc confirmed intracellular and intramitochondrial sulfur, which resulted in mitotoxic zinc sulfide (ZnS) precipitates. Together, GYY4137 increases intramitochondrial H_2_S and boosts oxygen consumption of endothelial cells, which is likely governed via the oxidation of H_2_S by SQOR. This mechanism in endothelial cells may be instrumental in regulating H_2_S levels in blood and organs but can also be exploited to quantify H_2_S release by soluble donors such as GYY4137 in living systems.

## 1. Introduction

Hydrogen sulfide (H_2_S) has antioxidant, anti-inflammatory, and anti-apoptotic properties that allow the prevention of cellular injury in various diseases, such as sepsis, ischemia-reperfusion, and neurodegenerative diseases [1,2,3,4]. One of the supportive effects of H_2_S is attributed to fuel mitochondria by donating electrons to the electron transport chain (ETC) after being oxidized by the sulfide:quinone oxidoreductase (SQOR) unit [5,6]. However, the physical and chemical properties of H_2_S make it a highly unstable molecule, whereby concentrations of H_2_S in biological matrices (e.g., blood plasma, cells) will drop within seconds following its administration by fast dispersion, evaporation, and reaction with proteins. Increasing the dosage of H_2_S will lead to higher levels in blood plasma, but the narrow therapeutic window, where H_2_S concentrations in the µM range and above lead to neurological dysfunction and rapid cardio-circulatory failure leading to cardiac arrest [7,8], preclude the use of H_2_S in higher dosages. Consequently, soluble long-acting H_2_S donors have been developed to provide sustained, physiologically relevant H_2_S levels in blood plasma. GYY4137 is a long-acting H_2_S donor that contains two sulfur groups producing H_2_S upon hydrolysis [9,10], demonstrated protective effects against oxidative stress, organ function, and apoptosis in animal models of sepsis, ischemia-reperfusion, arteriosclerosis, and neurogenerative diseases [10,11,12,13,14,15,16,17]. Thus, the narrow therapeutic window and volatile properties of H_2_S limit safe application in patients that can be overcome by the administration of H_2_S-donors to allow a safe release of H_2_S to govern cytoprotective effects and preserve organ function.

Although it is clear that GYY4137 can release H_2_S and protects against sepsis, ischemia-reperfusion, and neurodegenerative diseases [10,11,12,13,14,15,16,17], the mechanism by which GYY4137 supports mitochondrial function either via sulfhydration, metalloprotein interaction, antioxidant, or via direct H_2_S effects on the ETC is not elucidated yet [18]. As mitochondria play an important role in the pathophysiology of diseases in which H_2_S has a beneficial effect [5,6,16], intracellular H_2_S release by GYY4137 may be inferred to boost mitochondrial electron transport.

Endothelial cells likely comprise an important target of GYY4137, as they represent the primary target cell after parenteral administration. Moreover, endothelial dysfunction is a common hallmark of organ failure in sepsis, ischemia-reperfusion, and neurogenerative diseases [19,20,21,22]. To gain a better understanding of the mechanisms by which GYY4137 protects against organ failure in reaching mitochondria, it is essential to assess the release of H_2_S from GYY4137 within endothelial cells and its effect on cellular respiration.

The SQOR enzyme is able to oxidize H_2_S, yielding electrons to enter the mitochondrial electron transport chain. Most of the insight into the expression and function of SQOR comes from invertebrate species, while knowledge about the expression and function of SQOR in vertebrate species remains limited. The expression of SQOR differs between cell types, as intestinal cells have a relatively high expression, while the neuroblastoma cell line SH-SY5Y does not seem to express SQOR-like proteins [6,23,24]. In rats, SQOR is widely expressed, as shown in neurons, oligodendrocytes, endothelial cells, liver tissue, renal podocytes, tubular cells, sperm, and T cells [24,25]. The expression of SQOR is restricted to the mitochondria [24]. Remarkably, both cerebral and renal expression of SQOR increased during ageing in rats [24,25]. Whether human endothelial cells express SQOR is yet unknown.

We hypothesize that the presence of mitochondrial SQOR underlies the differential effects of GYY4137-derived H_2_S on mitochondrial oxygen consumption in different cell types. To this end, we confirmed the presence of SQOR in human umbilical vein endothelial cells (HUVECs) and quantified the direct functional effect of GYY4137 on SQOR by measuring mitochondrial oxygen consumption. Finally, we confirmed the ability of GYY4137-derived H_2_S to directly increase intramitochondrial H_2_S levels by trapping H_2_S with zinc. Collectively, these data demonstrate that GYY4137 influences mitochondrial function in endothelial cells depending on the presence of SQOR.

## 2. Materials and Methods

### 2.1. Cell Culture

HUVECs were obtained from the RuG/UMCG Endothelial Cell Facility. Briefly, primary isolates of umbilical cords were mixed and subsequently cultured on HUVECs culture medium, consisting of RPMI 1640 (Lonza, #BE12-115F, Breda, The Netherlands) supplemented with 20% heat-inactivated fetal calf serum (ThermoFisher Scientific, #10082147, Waltham, MA, USA), 2 mM l-glutamine (Life Technologies #25030, Carlsbad, CA, USA), 5 U/mL heparin (Leo Pharmaceutical Products, Amsterdam, The Netherlands), 1% Penicillin/Streptomycin (Sigma-Aldrich #P4333, St. Louis, MI, USA), and 50 μg/mL EC growth factor supplement from (Sigma-Aldrich, #E2759, St. Louis, MI, USA). The SH-SY5Y cells were used on DMEM culture media with 10% heat-inactivated fetal calf serum and 1% Penicillin/Streptomycin.

The cells were cultured in 75-cm2 tissue culture flasks (Corning #430720U, St. Louis, MI, USA) at 37 °C under 5% CO_2_/95% air. HUVECs were used for experiments up to passage 8. Cells were detached with trypsin (Sigma-Aldrich #25300, St. Louis, MI, USA). All compounds were dissolved in Milli-Q water. Cells were incubated with GYY4137 (Sigma-Aldrich, #SML0100, St. Louis, MI, USA) and or Zinc chloride (ZnCl_2_) (Sigma-Aldrich #3208086, St. Louis, MI, USA). Final concentrations of the mitochondrial inhibitors rotenone 1 µM (Sigma-Aldrich, R8875, St. Louis, MI, USA) and antimycin 5 µM (Sigma-Aldrich, #A8674, St. Louis, MI, USA).

### 2.2. Sulfide Solution, Preparation, and Use

A stock of 1 M sulfide solution was prepared from Na_2_S (Sigma-Aldrich) for each experiment. Ten microliters of this solution were diluted in 2 mL of Milli-Q water, and this 5 mM solution was immediately loaded in the glass syringes of the minipump so that it was not exposed to air for more than a few tenths of seconds. The pH of sulfide solutions was not equilibrated to a neutral value as it would enhance the volatility of sulfide by increasing H_2_S content.

### 2.3. Oxygen Consumption Rate Seahorse

Seahorse XF96 analyzers (Seahorse Biosciences, North Billerica, MA, USA) were used to assess the cellular oxygen consumption rate (OCR) and extracellular acidification rate (ECAR). Briefly, HUVECs were seeded in XF-96 cell culture plates (Seahorse Bioscience) at 1*10^4^ cells/well and incubated under standard conditions for 24 h. Cells were washed with XF Base RPMI (Seahorse Bioscience #103336, North Billerica, MA, USA) containing 8 mM glucose, 8 mM pyruvate, and 2 mM L-glutamine. The overall oxygen consumption rate was measured during the addition of GYY4137 (0.1 mM, 1 mM,10 mM)( Sigma-Aldrich, #SML0100, St. Louis, MI, USA) and ZnCl_2_ (Sigma-Aldrich #3208086, St. Louis, MI, USA). Experiments were conducted using six replicates for each condition and repeated in two independent experiments. Data were analysed by using Wave Desktop and Controller 2.6 Software.

### 2.4. Analysing the Presence of SQOR by Western Blot

Protein lysates were obtained using RIPA lysis buffer (50 mM Tris-Cl pH 8.0, 150 mM NaCl, 1% Igepal Ca 630, 0.5% Sodium Deoxycholate, 1.0% SDS, 0.4% protein inhibitor cocktail, 1 mM sodium orthovanadate, 10 mM NaF, 10 mM β-mercaptoethanol). Next, protein concentrations were measured with a Bio-Rad protein assay on a Bio-Tek Synergy H4 plate reader. Samples were loaded to 4–20% sodium dodecyl sulfate-polyacrylamide pre-casted gels (Bio-Rad TGX gels #4568096, Hercules, CA, USA) and transferred to a nitrocellulose membrane. A stain-free picture was captured to allow post-hoc normalisation for protein load. Membranes were blocked with 5% skimmed milk for 30 min and incubated with the primary antibody anti-SQORDL (Sigma-Aldrich #HPA017079, St. Louis, MI, USA) (1:1000, *v*/*v*) overnight at 4 °C. Secondary antibody goat anti-rabbit (DAKO #P0448, Santa Clara, CA, USA) (1:2000, *v*/*v*) were used to incubate for 2 hr at room temperature. Visualisation was performed using a SuperSignal (Perkin Elmer #NEL112001EA, Waltham, MA, USA) on a Bio-Rad ChemiDoc MP imaging system, while protein levels are quantified using ImageLab 6.0 (Bio-Rad, Hercules, CA, USA).

### 2.5. Measurement of Cellular Respiration and Sulfide Oxidation

The Oroboros O2k apparatus was used to monitor cellular oxygen consumption. Sulfide infusions or injections were made with the Tip2k minipump (Oroboros instrument). The respiration medium contained cell culture media. The pH was adjusted with 20 mM of HEPES buffer to a pH of 7.5. Cell suspensions were obtained after trypsinisation and immediately dissolved in culture media for measurements, for experimental procedures of GYY4137, 3 × 10^6^ cells/mL were used. Na_2_S experiments with HUVECs were performed with 1.5 × 10^6^ cells/ ml and SH-SY5Y with 3 × 10^6^ cells/mL.

### 2.6. Imaging

HUVECs were cultured on coverslips of glass, coated with gelatin 2%, and incubated with GYY4137 for 30 min. The TMRM (100 nM) (Thermofisher #T668, Waltham, MA, USA) and AzMC (10 µM) (Sigma-Aldrich #802409, St. Louis, MI, USA) were incubated for 20 min and washed with HBSS. Images were measured with the Deltavision Elite microscope emission/excitation filter settings DAPI/FITC for AzMC and TRITC/TRITC for TMRM. Fluorescence was analysed with ImageJ.

### 2.7. AzMC Dose Response

Cells were cultured in a 96-wells plate until confluent, then the culture medium was replaced by HBSS supplemented with Na_2_S or GYY4137 and loaded with AzMC (10 µM). Fluorescence was measured after 30 min with the Bio-Tek Synergy H4 plate reader at ex: 340 and em: 445.

### 2.8. Lead Acetate

Lead acetate paper reacts with hydrogen sulfide and forms brownish-black lead sulfide. It is prepared by soaking filter paper in a 1% lead acetate solution followed by drying. Cells were cultured in a 96-wells plate, covered with lead acetate papers, and placed at 37 °C under 5% CO_2_/95% air. Images were taken with ChemiDoc and analysed with ImageJ.

### 2.9. Electron Microscopy

HUVECs were cultured in a 24-wells plate. GYY4137, zinc, and GYY4137 together with zinc were added, and after 30 min incubation, cells were embedded. HUVECs were fixed with 2%/2% formaldehyde/glutaraldehyde for 30 min at room temperature. Cells were washed 3 × in 0.1 M PBS. A second fixation was performed in 1% osmium tetroxide in 0.1 M PBS for 1 h at 4 °C. After washing (3 times in water), cells were gradually dehydrated in graded series of ethanol and then gradually infiltrated with resin at room temp. Gelatin capsules were put upside-down on the coverslips and polymerised for 24 h at 60 °C. Samples were lightly heated to remove the glass coverslip and cut on an ultramicrotome Reichert S at 90 nm of thickness. Acquisitions were performed on a JEOL 1011 TEM with a Gatan Orius 1000 CCD Camera.

### 2.10. Data Analysis

Statistical analysis was performed using a GraphPad Prism 7.02 for Windows. Kruskal-Wallis test, followed by a Mann-Whitney U-test in the case of non-normally distributed variables, was used to calculate statistical differences between groups. For normally distributed variables, ANOVA was used. Data are expressed as mean ± standard error of the mean. *p* < 0.05 was considered statistically significantly different. Images were created with BioRender.com.

## 3. Results

### 3.1. Human Umbilical Vein Endothelial Cells Oxidize H_2_S via the Sulfide:Quinone Oxidoreductase

**Figure 1:**
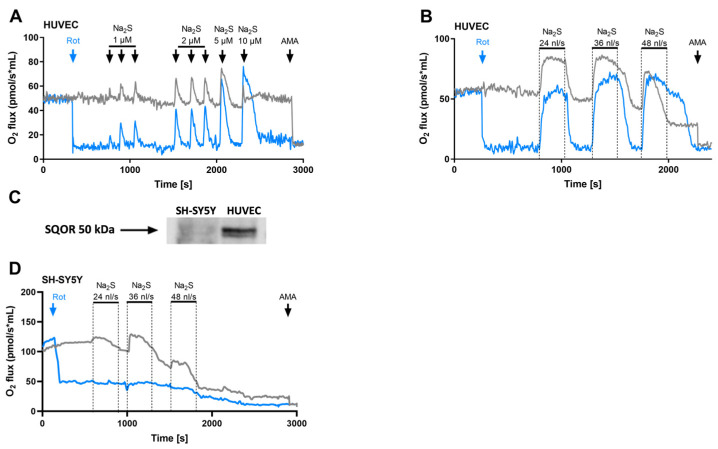
Sodium sulfide increased oxygen consumption in HUVEC but not in SH-SY5Y cells. Cells were introduced in the two chambers of the oxygraphy (O2k). (**A**) HUVEC were incubated with a bolus of sodium sulfide (Na_2_S; final concentrations in the medium are shown in figure), which was repeated upon co-incubation with rotenone (Rot) 1 µM to inhibit electron transfer via complex I, Antimycin A (AMA) 5 µM was added as the control to inhibit mitochondrial electron transfer via complex III. (**B**,**C**) HUVEC and SH-SY5Y cells upon continuous infusion of 5 mM Na_2_S (infusion rates are shown in figure), which was repeated upon co-incubation with rotenone (Rot) 1 µM to inhibit electron transfer via complex I, AMA 5 µM was added as the control to inhibit mitochondrial electron transfer via complex III. (**D**) Protein level of sulfide:quinone oxidoreductase (SQOR) in SH-SY5Y cells and HUVEC. AMA; antimycin A, HUVECs; human umbilical vein endothelial cells, Rot; Rotenon, SQOR: sulfide:quinone oxidoreductase.

Cellular H_2_S oxidation is accomplished by SQOR, a protein part of the sulfide oxidation unit (SOU), together with the sulfur dioxygenase and the thiosulfate-cyanide sulfurtransferase, catalysing sulfide oxidation to thiosulfate [6,23]. Consequently, cellular H_2_S oxidation is characterized by two properties: firstly, in contrast with carbon oxidation (Krebs cycle), it is resistant to inhibition by rotenone. Secondly, sulfide oxidation by SQOR requires oxygen, hence it increases cellular oxygen consumption. Therefore, in HUVECs, the presence of SQOR activity will be measured [6]. In agreement with sulfide oxidation by SQOR, administration of sodium sulfide (Na_2_S, 1–5 µM) dose-dependently increased oxygen consumption of HUVECs (Figure 1A grey trace), which effect was also observed when the endogenous respiration was inhibited by rotenone (inhibition of complex I and the Krebs cycle), uncovering the electron entrance via SQOR (Figure 1A, blue trace). While both traces (Figure 1A) revealed similar kinetics over the 1–5 µM Na_2_S range, a sharp difference appeared when the final Na_2_S concentration reached 10 µM, presumably due to partial inhibition of mitochondrial complex IV by sulfide. Thus, the dose-dependent increase of oxygen consumption by H_2_S supports the presence of an SQOR in endothelial cells.

Next, to quantify the oxidation capacity of SQOR, we used a continuous infusion of sulfide in the absence and presence of rotenone, resulting in a synchronous increase in cellular oxygen consumption when the 5 mM solution of Na_2_S was infused at a rate of 24 nL/s (Figure 1B). Increasing the infusion rate to 36 nL/s disrupted this similarity between the oxygen consumption in the control group (grey trace) and the rotenone group (blue trace), explained by competing electrons entrance via complex I from the Krebs cycle in control cells. While a further increase of Na_2_S infusion rate to 48 nL/s persistently inhibited oxygen consumption in HUVEC, it increased oxygen consumption in the presence of rotenone, which was maintained after the cessation of infusion. This observation indicates a saturation of SQOR activity with an accumulation of H_2_S, which in the presence of rotenone remains available for oxidation after the infusion, while in the absence of rotenone results in inhibition of cellular respiration. To substantiate that SQOR function in HUVECs is unaffected by passaging of cells, experiments were repeated in HUVECs within 2 h after their isolation from the umbilical cord. Freshly isolated HUVECs showed a similar sulfide oxidation capacity as HUVECs (Figure S1). In addition, HUVECs express detectable protein levels of SQOR, while SH-SY5Y cells do not express detectable levels (Figure 1C and Figure S1B). Consistently, SH-SY5Y cells without SQOR failed to increase oxygen consumption upon Na_2_S, either in the presence or absence of rotenone (Figure 1D). Moreover, the continuous infusion of sulfide produced a gradual inhibition of oxygen consumption. Finally, antimycin A blocked mitochondrial complex III, and thereby mitochondrial oxygen consumption (Figure 1B grey trace), demonstrating electron donation upstream to complex III of the mitochondrial electron transport chain. Together, these results demonstrate the presence of a potent SQOR in endothelial cells and argue for the importance of endothelial cells in regulating H_2_S levels in blood and organs.

### 3.2. Stoichiometry between H_2_S Oxidation and Oxygen Consumption

**Figure 2:**
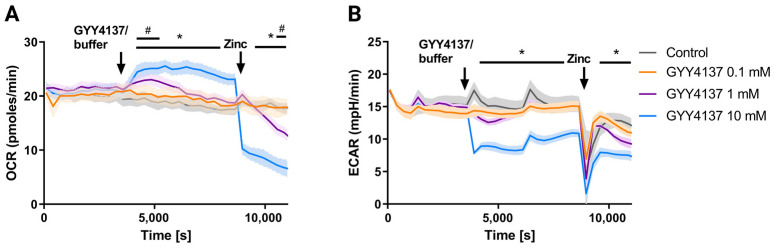
GYY4137 dose-dependently increased oxygen consumption rate in HUVECs, which was blocked by co-incubation with zinc chloride. (**A**) Oxygen consumption rate (OCR) in HUVECs incubated with 0.1 mM, 1 mM, and 10 mM GYY4137 or a buffer medium (control), followed by incubation with 800 µM zinc chloride (zinc). (**B**) Extracellular acidification rate (ECAR) in HUVECs incubated with 0.1 mM, 1 mM, and 10 mM GYY4137 or a buffer medium (Con), followed by incubation with 800 µM zinc chloride (zinc). * *p* < 0.05 between GYY4137 10 mM and control, # *p* < 0.05 between GYY4137 1 mM and control. ECAR; extracellular acidification rate. HUVECs; human umbilical vein endothelial cells, OCR: oxygen consumption rate. Data are represented as mean ± SEM, * means *p* < 0.05.

Sulfide Oxidation by SQOR and endogenous respiration, the stoichiometry between the sulfide infusion rate and the increase in oxygen consumption rate was assessed (∆JO_2_) (Table 1). Basal respiration of HUVECs (1.5 × 10^6^/mL) amounted 55 O_2_ pmol/(s × mL) (Figure 2B). In this experiment, the maximal rate for SQOR activity in the HUVECs was able to neutralise 24 nL/s of Na_2_S (5 mM) in the 2 mL chamber, hence a sulfide flux of (24 × 5 ÷ 2) = 60 pmol/(s × mL). Rotenone reduced basal oxygen consumption to 10 pmol O_2_/(s × mL), and infusion of Na_2_S raised it to 40 pmol/(s × mL). The theoretical stoichiometry O_2_/Na_2_S for oxidation of sulfide is 1.0, and the difference with the experimental value observed here in the presence of rotenone (40/60 = 0.67) results, for the largest part, from impure/degraded sulfide with less than the theoretical sulfide concentration in the solution infused. When sulfide infusion took place in the presence of the endogenous respiration, the presence of SQOR ensured the same sulfide elimination rate, but the increase in cellular oxygen consumption was only 28 pmol/(s × mL). The difference is expected to reveal interactions between SQOR and endogenous respiration and notably with complex I., the target of rotenone inhibition. Accordingly, if one assumes an unchanged stoichiometry of sulfide oxidation by SQOR in the absence and presence of rotenone, the difference (40 − 28 = 12 pmol O_2_/(s × mL)) represents the reduction in complex I activity caused by SQOR. Half of the oxygen consumption rate observed with sulfide in the presence of rotenone is explained by electron transfer in the mitochondrial electron transport chain and cytochrome oxidase reaction. The other half is explained by the dioxygenase activity of SOU. With the maximal rate of 40 pmol O_2_/(s × mL) oxygen consumption, 20 resulted from cytochrome oxidase reaction. This is to be compared with the endogenous respiratory rate that recruited cytochrome oxidase oxygen consumption at a rate of 55. The experimental stoichiometries in this report are close to others values reported so far with measurements made in similar conditions. It makes it then very likely that here we observe the activity of SOU that is expected to be present in the majority of mammalian cell lines. Hence when artificially recruited by high sulfide levels, the SQOR present in HUVECs cells could ensure a consequent activity of the mitochondrial respiratory chain (at 40% of the normal respiratory rate). 

### 3.3. SQOR Oxidises H_2_S Released from GYY4137

Because of the fast evaporation of H_2_S, Na_2_S effects on oxygen consumption cannot be assessed using the Seahorse apparatus due to its open architecture [6]. Yet, in contrast, Seahorse can be used to assess the effects of GYY4137, being a slow-releasing H_2_S donor, with the advantage of increasing cell/medium ratio [from 1.5–3 × 10^3^ cells per µL (Oroboros) to 1 × 10^4^ cells per µL (Seahorse). GYY4137 (0.1–10 mM) dose-dependently increased cellular oxygen consumption in HUVECs (Figure 2A). Next, we sought to scavenge H_2_S released from GYY4137 by the addition of an excess of zinc ions, which remove sulfide ions from the solution because of the low solubility of zinc sulfide (ZnS; ~10^−25^ *K_sp_*) (Figure S2). The addition of zinc ions dose-dependently decreased GYY4137-induced oxygen consumption (Figure 2A), but the effect appeared to extend beyond the normalisation of the oxygen consumption back to the value obtained before the GYY4137 injection. Furthermore, the extracellular acidification rate acutely dropped upon the administration of the highest concentration of GYY4137, which is likely caused by the abrupt pH change upon injection of the GYY4137 solution (Figure 2B). Collectively, these data show that GYY4137 released H_2_S increased cellular respiration of SQOR-expressing HUVECs.

### 3.4. GYY4137 Only Increases Oxygen Consumption in Cells with a SQOR

**Figure 3:**
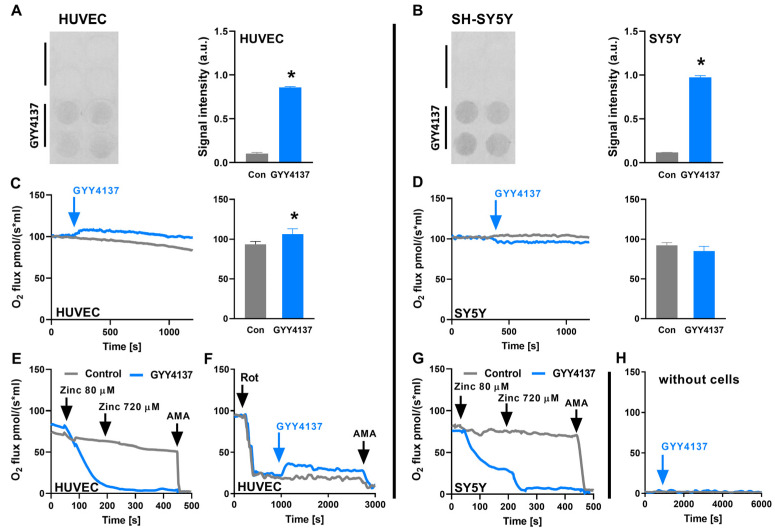
GYY4137 released intracellular H_2_S and donated electrons at the electron transport chain in HUVEC, but not SH-SY5Y cells. (**A**) H_2_S release by GYY4137 in HUVECs and (**B**) SH-SY5Y after 48 h, measured using lead acetate papers (*n* = 4/group). (**C**) Oxygen consumption of HUVECs and (**D**) SH-SY5Y treated with GYY4137 (blue arrow) in cell culture medium (*n* = 3/group). (**E**) Incubation of HUVEC with 10 mM GYY4137 (blue line) followed by 80 µM and 800 µM zinc chloride (zinc) inhibited oxygen consumption in the presence of GYY4137. (**F**) Oxygen consumption of HUVECs treated with rotenone (Rot) followed by incubation with GYY4137 or solvent (arrow), finally antimycin A (AMA) 5 µM was added to inhibit mitochondrial oxygen consumption. (**G**) In SH-SY5Y cells administration of GYY4137 10 mM (blue line), followed by adding 80 µM and 800 µM zinc inhibited oxygen consumption in the presence of GYY4137, finally AMA 5 µM was added to inhibit mitochondrial oxygen consumption. (**H**) In the absence of cells, GYY4137 10 mM was added and oxygen consumption was measured. AMA; antimycin A. HUVECs; human umbilical vein endothelial cells, Rot; rotenone, SY5Y; SH-SY5Y. Data are represented as mean ± SEM, * means *p* < 0.05.

To assess H_2_S release by GYY4137 in HUVECs with SQOR and SH- SY5Y without SQOR, we measured the sulfide released by cells using lead acetate paper placed on top of cell culture wells. GYY4137 (10 mM; 48 h) blackened lead acetate paper, signifying H_2_S release in HUVECs and SH-SY5Y cells (Figure 3A,B). Next, we estimated the H_2_S release from GYY4137 by measuring oxygen consumption in cells using the closed 1 mL chamber of the Oroboros. Administration of GYY4137 (10 mM) increased oxygen consumption in HUVEC by 12.7 pmol O_2_/(s × mL) (Figure 3C), while the effect, if any, would be a slight decrease that remained non-significant with SH-SY5Y cells lacking SQOR (Figure 3D). Administration of 80–800 µM zinc chloride (black arrow) to HUVECs inhibited oxygen consumption only in the presence of GYY4137 (blue trace; Figure 3E), while zinc chloride was without effect on cellular oxygen consumption in control cells. Next, to validate SQOR activity, HUVECs were treated with rotenone to inhibit complex I and the Krebs cycle incubated with GYY4137 (10 mM), which increased oxygen consumption initially with 14 pmol O_2_/(s × mL) (Figure 3F). Administration of 80–800 µM zinc chloride (black arrow) to SH-SY5Y cells inhibited oxygen consumption only in the presence of GYY4137 (blue trace; Figure 3G), while zinc chloride was without effect on cellular oxygen consumption in control cells. As expected, oxygen consumption was absent in GYY4137 administered to the culture medium (Figure 3H). The increase in oxygen consumption caused by GYY4137 in the presence of rotenone could be considered to result from SQOR activity and be proportionate to the H_2_S release by GYY4137. The theoretical oxygen-to-sulfide stoichiometry of one for the SOU reaction results in the same value for oxygen consumption and sulfide release. Thus, high levels of GYY4137 release a detectable amount of H_2_S on mitochondrial SQOR, providing insight into the releasing capacity following the administration of this sulfide donor. Together, GYY4137 released H_2_S in HUVECs and SH-SY5Y cells, yet its effect on mitochondrial oxygen consumption is only observed in HUVEC, which is likely explained by the presence of SQOR.

### 3.5. GYY4137 Increased Intramitochondrial H_2_S Levels

**Figure 4:**
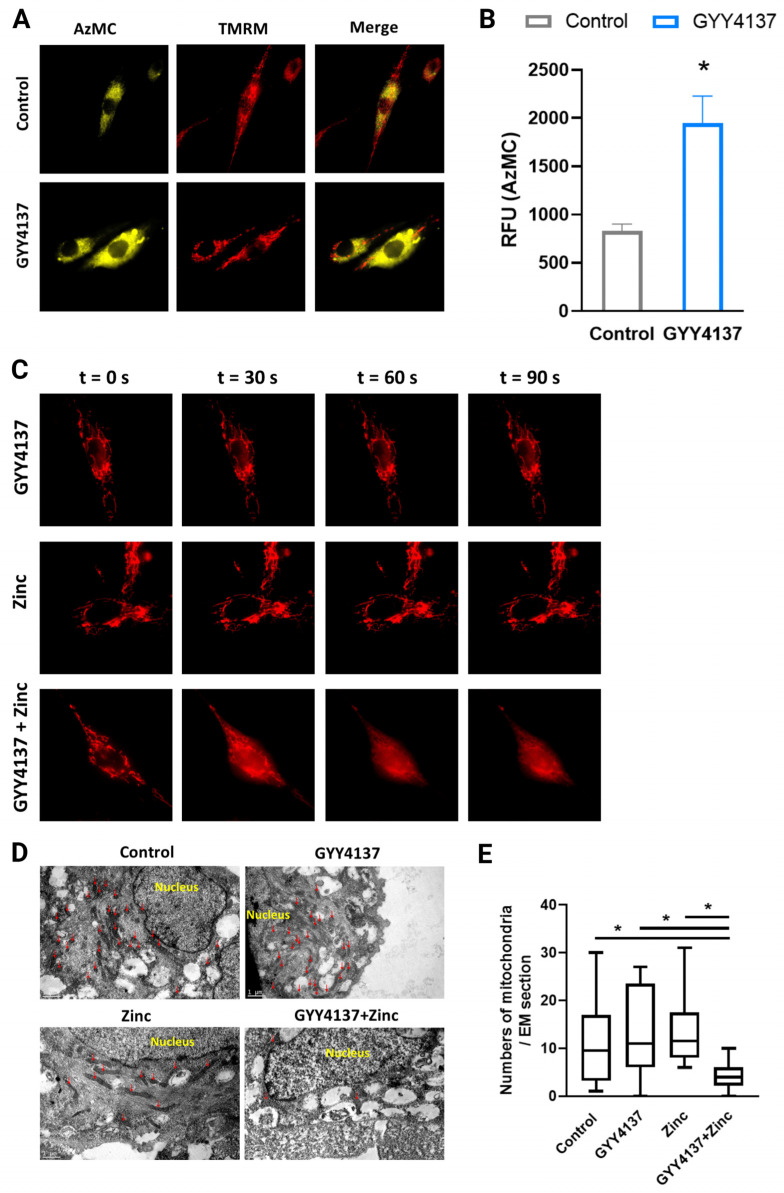
GYY4137 leads to increased intra-mitochondrial levels of hydrogen sulphide. (**A**) HUVECs incubated with GYY4137 for 30 min were stained with AzMC (yellow) and mitochondria with TMRM (red) and merged, (**B**) followed by the quantification of AzMC (*n* = 8/group). Representative images were captured at 60× magnification. (**C**) HUVECs incubated for 30 min with GYY4137, zinc chloride (zinc), or zinc after 30 min GYY4137, mitochondria were stained with TMRM (red). Representative images were captured at 60× magnification. (**D**) Electron microscopy images of HUVECs without treatment and HUVECs treated with 800 µM zinc, 10 mM GYY4137, or both (GYY4137 + zinc), red arrows point to mitochondria. Scale bar: 1 μm. (**E**) Number of mitochondria per field in electron microscopy (at least 20 fields were quantified). AzMC; 7-Azido-4-methylcoumarin, EM; Electron microscopy, HUVECs; human umbilical vein endothelial cells, TMRM; tetramethyl rhodamine methyl ester. Data are represented as mean ± SEM, * means *p* < 0.05.

Next, we assessed intracellular H_2_S levels in HUVECs that were loaded with the 7-azido-4-methyl-coumarin (AzMC) fluorescent H_2_S-probe and the mitochondrial fluorophore tetramethylrhodamine methyl ester (TMRM) (Figure 4A,B and Figure S3A). GYY4137 demonstrated widespread H_2_S release in organelles and overlapping in mitochondria. Given that GYY4137 releases intracellular H_2_S and that zinc co-administration inhibited mitochondrial oxygen consumption, we next assessed the mitochondrial membrane potential with TMRM in HUVECs treated with GYY4137, zinc chloride, and the combination of both (Figure 4C). While the treatment with GYY4137 or zinc chloride alone did not affect the mitochondrial membrane potential, co-treated cells showed an immediate drop in mitochondrial membrane potential. In addition, mitochondrial morphology was assessed for the same conditions with electron microscopy (Figure 4D,E), whereas mitochondrial numbers were unaffected by treatment with GYY4137 or zinc chloride alone, co-treated cells showed a decrease in mitochondrial number (Figure 4E and Figure S3B,C), in line with the observed decrease in cellular oxygen consumption under these conditions (Figure 2A). Thus, H_2_S release from GYY4137 is widely distributed inside HUVECs, including mitochondria, and together with zinc, leads to the formation of mito-toxic zinc-sulfide precipitates, resulting in a reduced number of mitochondria.

## 4. Discussion

### 4.1. Sulfide Oxidation by Human Endothelial Cells

Hydrogen sulfide is widely used as a therapeutic intervention to improve disease outcomes in experimental models. To translate the experimental use of H_2_S donors into clinical applications, the processing of H_2_S inside the cell must be explored to understand the final distribution for dosing. Endothelial cells constitute the first barrier for parenteral administered circulating H_2_S and H_2_S-donors to reach any end organ. The present study demonstrates that human endothelial cells express a functional SQOR. Maximal stimulation of SQOR in HUVECs had the consequence that electrons from H_2_S accounted for up to 40% of total electron flow in the respiratory chain. We further eliminated the possibility of SQOR expression in HUVECs to be artefactual and resulting from cell culture conditions, as similar responses were found in freshly isolated cells. In addition, the widely used H_2_S-donor GYY4137 was assessed via SQOR in human endothelial cells to measure actual H_2_S release, demonstrating a dose-dependent (1 mM–10 mM) increase in oxygen consumption. The H_2_S release of GYY4137 increases oxygen consumption in HUVECs (which contain an SQOR), while this effect was absent in SH-SY5Y cells (lacking an SQOR). Furthermore, co-incubation of cells with GYY4137 and zinc chloride leads to the formation of mito-toxic zinc-sulfide precipitates as observed by electron microscopy and leads to mitochondrial loss, thereby confirming relevant intramitochondrial levels of H_2_S derived from GYY4137. Together, we demonstrated endothelial cells to scavenge H_2_S by SQOR, which allowed us to assess the H_2_S release of GYY4137 on the mitochondria of human endothelial cells.

### 4.2. H_2_S Donation by GYY4137 Influences Mitochondrial Oxygen Consumption

Despite the beneficial effects of GYY4137, the H_2_S amount released by GYY4137 remains unclear in living systems. The intracellular H_2_S release rate of GYY4137 has been questioned by studies showing different release rates and measured outside the cell, showing GYY4137 concentration of 1 mM releasing H_2_S levels ranching from 2 µM to 100 µM [9,26,27,28,29,30,31]. The assays used so far to assess H_2_S-release by GYY4137 in vitro, such as the methylene blue method, influence acidity, thereby shifting the equilibrium towards H_2_S outside the living system, questioning its relevance for assessing intracellular H_2_S [17].

Under physiological conditions, the endogenous flow of sulfide release is expected to be considerably lower than the flux required for this maximal stimulation of SQOR [32]. Consequently, under physiological conditions, SQOR operates well below its maximal rate, hence with a large enzymatic reserve able to avert sulfide accumulation. Moreover, it indicates intracellular sulfide concentrations that would remain far below the SQOR affinity constant (K_m_) for sulfide, which is around 1 µM [32,33]. To demonstrate H_2_S release from GYY4137 to increase H_2_S levels inside mitochondria, we administered zinc ions. The toxicity of zinc for cellular respiration in the presence of GYY4137 is expected to result from the intracellular formation of ZnS and constitutes a further argument for the intracellular release of sulfide from GYY4137. Two factors could cooperate to enhance mitochondrial sensitivity to ZnS formation: mitochondrial accumulation of the Zn^2+^ driven by its membrane potential and/or intramitochondrial generation of sulfide. Altogether, GYY4137 demonstrates that measurable H_2_S release takes place within cells, which is removable by zinc ions forming the highly insoluble zinc sulfide (ZnS).

Nevertheless, the sulfide release from GYY4137 is slow, necessitating mM concentrations of GYY4137 to detect SQOR activity in acute experiments. The present study clarifies this issue by demonstrating unambiguously that GYY4137 administration to cells causes a direct increase in mitochondrial oxygen consumption with characteristics fully consistent with the mitochondrial sulfide oxidation by SQOR.

### 4.3. GYY4137 Administration and Consequences of Intracellular (Autocrine) H_2_S Release

Here, we provided insight into the ability of GYY4137 to release H_2_S, resulting in increased oxygen consumption. The slow-release rate of sulfide by GYY4137 is expected to generate a steady state with a low sulfide concentration in cells. The steady-state concentration would result from the balance between sulfide generation and elimination rates with a prominent role for SQOR, if present, in elimination. Therapeutic effects are expected to originate from the sum of endogenous and GYY4137-derived sulfide release, generating an increased steady-state concentration of sulfide, in turn resulting in an oxygen-dependent effect on mitochondrial bioenergetics and/or stimulation of diverse sulfide signaling pathways [32]. However, it should be mentioned that hypoxia leads to increased production of endogenous H_2_S and that inhibiting sulfide-producing enzymes or chemically scavenging sulfide has also demonstrated protective effects against ischemia/reperfusion, comparable to the administration of exogenous sulfide donors. SQOR may exert its protective effects by scavenging H_2_S, as sulfide pre-conditioning in mice by breathing H_2_S led to the upregulation of SQOR and made the mice more tolerant to hypoxia [34].

Given that endothelial cells are the first barrier to pass for plasma-bound GYY4137, it is essential to assess whether SQOR processes sulfide that is intra or extracellularly generated from GYY4137-released H_2_S in endothelial cells by direct measurement of mitochondrial SQOR activity. Although we demonstrate the direct effects of GYY4137-derived H_2_S on mitochondrial oxygen consumption of endothelial cells, it remains unknown to what extent these effects of GYY4137-derived H_2_S are mediated by its direct effects on endothelial cells or whether GYY4137 also diffuses into target tissues. To answer this question, future pharmacokinetic studies would be needed to assess the distribution of GYY4137 into target tissues. Until then, we should realize that the beneficial effects of parenteral-administered H_2_S-donors such as GYY4137 are likely mediated by their effect on endothelial cells. Further, genetically silencing SQOR would allow excluding alternative pathways that may contribute to H_2_S catabolism in human endothelial cells.

### 4.4. Effects of H_2_S on Protein Function

Protein activity can be affected by post-translational modification. H_2_S can modify proteins post-translationally through a process called persulfidation, which affects protein activity, localisation, and interactions with other proteins. SQOR and thiosulphate sulfurtransferase (TST) are enzymes that produce persulfides (RSSH) during H_2_S oxidation and thereby can affect post-translational protein modifications [35]. Persulfidation mainly occurs on cysteine residues, preventing them from being oxidized by free radicals and allowing the preservation of protein function [36]. The availability of H_2_S for persulfidation depends on cellular redox status, and during oxidative stress, the number of substrates such as cysteine for H_2_S production may be limited [37]. Furthermore, the indirect effect of H_2_S can affect protein function. As such, the reduction of intramitochondrial Fe^3+^ to Fe^2+^ that is catalyzed by H_2_S affects the function of cytochrome *c* and phosphodiesterase proteins [38]. To our knowledge, whether persulfidation affects SQOR activity is yet unknown.

## 5. Conclusions

In endothelial cells, the release of H_2_S via GYY4137 increases mitochondrial H_2_S levels. Because endothelial cells possess an SQOR, GYY4137-released sulfide increases mitochondrial oxygen consumption, which is absent in SH-SY5Y cells without SQOR. H_2_S can increase the total mitochondrial electron flow in endothelial cells by up to 40% in the presence of SQOR. Our results are of relevance to understanding the in vivo pharmacologic effects and the key role of endothelial cells herein of soluble H_2_S donors such as GYY4137.

## Figures and Tables

**Table 1:** Oxygen consumption rate of HUVECs in the presence of sodium sulfide. Oxygen flux in HUVEC is demonstrated for the different infusion rates of sodium sulfide (Na_2_S) and with 1 µM rotenone to inhibit complex I and the Krebs cycle. The stoichiometry was assessed at an infusion rate of 24 nL/s Na_2_S. Oxygen consumption rate was measured as the maximum respiration was reached within seconds after the start of infusion. ∆JO_2_: change in oxygen consumption rate as compared to baseline in pmol/s × mL, JNa_2_S: sulfide infusion rate in nL/s.

	Baseline	24 nL/s Na_2_S	36 nL/s Na_2_S	48 nL/s Na_2_S	Stoichiometry @24 nL/s
	O_2_ pmol/s × mL	O_2_ pmol/s × mL	O_2_ pmol/s × mL	O_2_ pmol/s × mL	∆JO_2_/JNa_2_S
Control	55	83	83	72	0.47
Rotenone	10	50	68	68	0.67

## Data Availability

The datasets generated during and/or analysed during the current study are available from the corresponding author upon reasonable request.

## Supplementary Materials

The following supporting information can be downloaded at: https://www.mdpi.com/article/10.3390/antiox12030587/s1. Figure S1. Freshly isolated HUVECs demonstrated activity of the sulfide oxidation unit and stain free blot of HUVEC and SH-SY5Y. (A) Oxygen consumption of freshly isolated HUVECs (grey) upon incubation with the fast H2S donor Na2S in freshly isolated HUVECs (grey) as compared to empty chamber with culture medium (blue). (B) Stain free blot of the SQOR stained membrane with the lanes respectively: ladder, SH-SY5Y cells and HUVECs. Figure S2. Zinc chloride reacts with hydrogen sulfide from Na_2_S or GYY4137 and forms zinc sulfide precipitates. Left: 800 μM Zinc chloride (zinc) in water added to 1 mM Na_2_S in water; right: 800 μM zinc added to 10 mM GYY4137 in water. Figure S3. Mitochondria and vesicles in HUVECs after incubation with zinc chloride, GYY4137 or both combined. (A) Cumulative AzMC fluorescence after 30 minutes with Na2S or GYY4137 in HU-VECs. (B) Electron microscopy images of HUVECs with 10 mM GYY4137, 800 μ M zinc chloride, control and both combined. (C) Quantification of mitochondria, vesicles and their ratio (at least 20 fields were used for quantification). Red arrows: mitochondria, blue arrows: vesicles. Data are rep-resented as mean ± SEM, * means *p* < 0.05.
