# Supplementary material for: GYY4137-Derived Hydrogen Sulfide Donates Electrons to the Mitochondrial Electron Transport Chain via Sulfide: Quinone Oxidoreductase in Endothelial Cells
Source: Antioxidants (Basel). 2023 Feb 27;12(3):587. doi: 10.3390/antiox12030587 (PMC10044827; doi:10.3390/antiox12030587)
Supplement: Supplementary file 1 [file antioxidants-12-00587-s001.zip › antioxidants-2211576-supplementary.pdf]

Supplementary Materials: The following supporting information can be downloaded at: www.mdpi.com/xxx/s1,

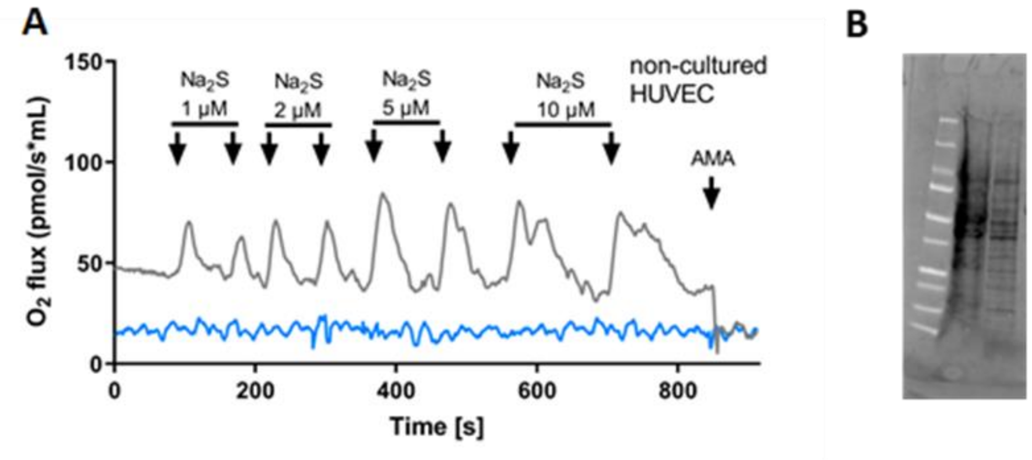

Figure S1. Freshly isolated HUVECs demonstrated activity of the sulfide oxidation unit and stain free blot of HUVEC and SH-SY5Y. (A) Oxygen consumption of freshly isolated HUVECs (grey) upon incubation with the fast H_2S donor Na_2S in freshly isolated HUVECs (grey) as compared to empty chamber with culture medium (blue). (B) Stain free blot of the SQOR stained membrane with the lanes respectively: ladder, SH-SY5Y cells and HUVECs.

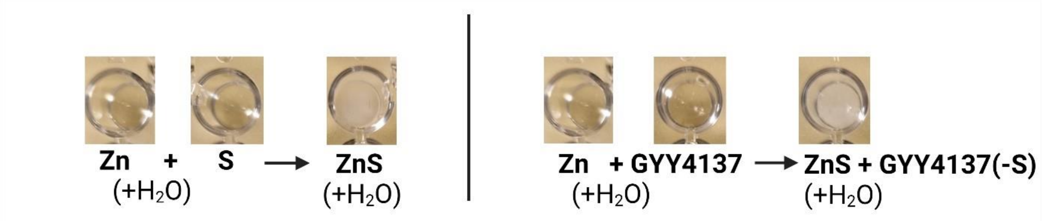

Figure S2. Zinc chloride reacts with hydrogen sulfide from Na_2S or GYY4137 and forms zinc sulfide precipitates. Left: 800 µM Zinc chloride (zinc) in water added to 1 mM Na_2S in water; right: 800 µM zinc added to 10 mM GYY4137 in water.

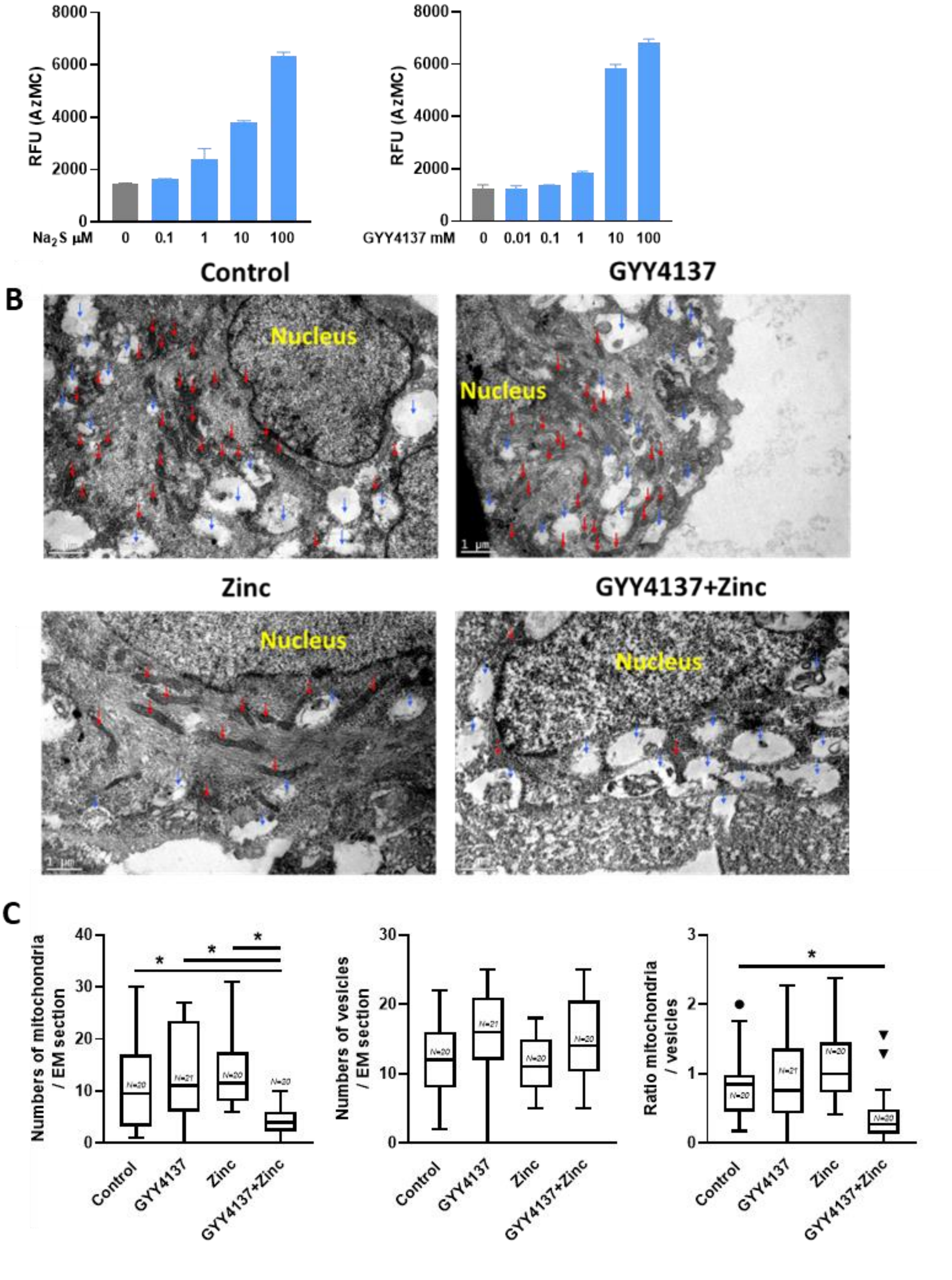

Figure S3. Mitochondria and vesicles in HUVECs after incubation with zinc chloride, GYY4137 or both combined. (A) Cumulative AzMC fluorescence after 30 minutes with Na_2S or GYY4137 in HUVECs. (B) Electron microscopy images of HUVECs with 10 mM GYY4137, 800 μM zinc chloride, control and both combined. (C) Quantification of mitochondria, vesicles and their ratio (at least 20 fields were used for quantification). Red arrows: mitochondria, blue arrows: vesicles. Data are represented as mean ± SEM, * means $p < 0.05$.
